# Green synthesis of binary FeOOH/Fe_2_O_3_ nanosized composite using *Leucaena leucocephala* seeds and their effect on mung bean under drought stress

**DOI:** 10.1186/s12870-026-08421-0

**Published:** 2026-03-20

**Authors:** Eman M.M. Eldebawy, Eman G. El-Hosary, Amel F. Elhusseiny, Eman Hassan Elsayed, Mona E. M. Mabrouk, Salwa M. Abdel Rahman

**Affiliations:** 1 Botany and Microbiology Department, Faculty of Science, Damanhour University, Damanhour, Egypt; 2 Department of Pharmaceutical Production Technology, Faculty of Applied Health Sciences, Borg Al Arab Technological University, Alexandria, Egypt; 3 Department of chemistry, Faculty of Science, Alexandria University, Alexandria, Egypt; 4 Department of Botany and Microbiology, Faculty of Science, Alexandria University, Alexandria, Egypt

**Keywords:** Binary nanosized composite, Drought stress, Mung bean, Green chemistry, Sustainable agriculture

## Abstract

**Background:**

Drought is widely acknowledged as one of the most important abiotic stresses affecting crop productivity worldwide. In this context, a green, facile and low-cost method using *Leucaena leucocephala* seeds extracts and iron sulfate was used to synthesize a a binary FeOOH/Fe_2_O_3_ nanosized composite (BFNC) and investigated it as a nanoprimer for mung bean cultivation under drought stress.

**Methods:**

A green and facile and low-cost method using *Leucaena leucocephala* seeds extracts and iron sulfate was used to synthesize the BFNC, which was characterized by XPS, FT-IR, UV-Vis, EDX, XRD, SEM, TEM, and Zeta potential analysis. A pot experiment was conducted to investigate the effect of seed priming with different aqueous BFNC solution (5, 10, 20 mg/L) for 6 h on mung bean under drought stress.

**Results:**

Based on the binding energies at 712.28 eV in the Fe2p3/2 region and the distinguishing multiple shake up satellite structures, the XPS analysis revealed that iron was predominantly present in the Fe^3+^ oxidation state and the coexistence of FeOOH and Fe_2_O_3_ phases, forming a BFNC. The FT-IR results indicated that the BFNC was capped well with the plant extract. The SEM image showed a well-separated spherical nanoparticle morphology with asize range from 13 to 18 nm. The study findings revealed that seed priming with 5 mg/L BFNC was the most effective treatment for enhancing growth parameters and biochemical attributes of mung bean under drought stress.

**Conclusions:**

The use of the green-synthesized BFNC is a promising, and sustainable approach to mitigate the damaging effects of drought on agricultural crops. whereas the, seeds extract serves as an excellent oxidizing, caping and, stabilizing agent. However, as reported in most of the literature the green synthesis of iron based nanoparticles, utilized plant extract, as a bio reduction agent.This practice directly supports the Sustainable Development Goals (SDGs) related to climate action and ensuring global food security.

**Graphical Abstract:**

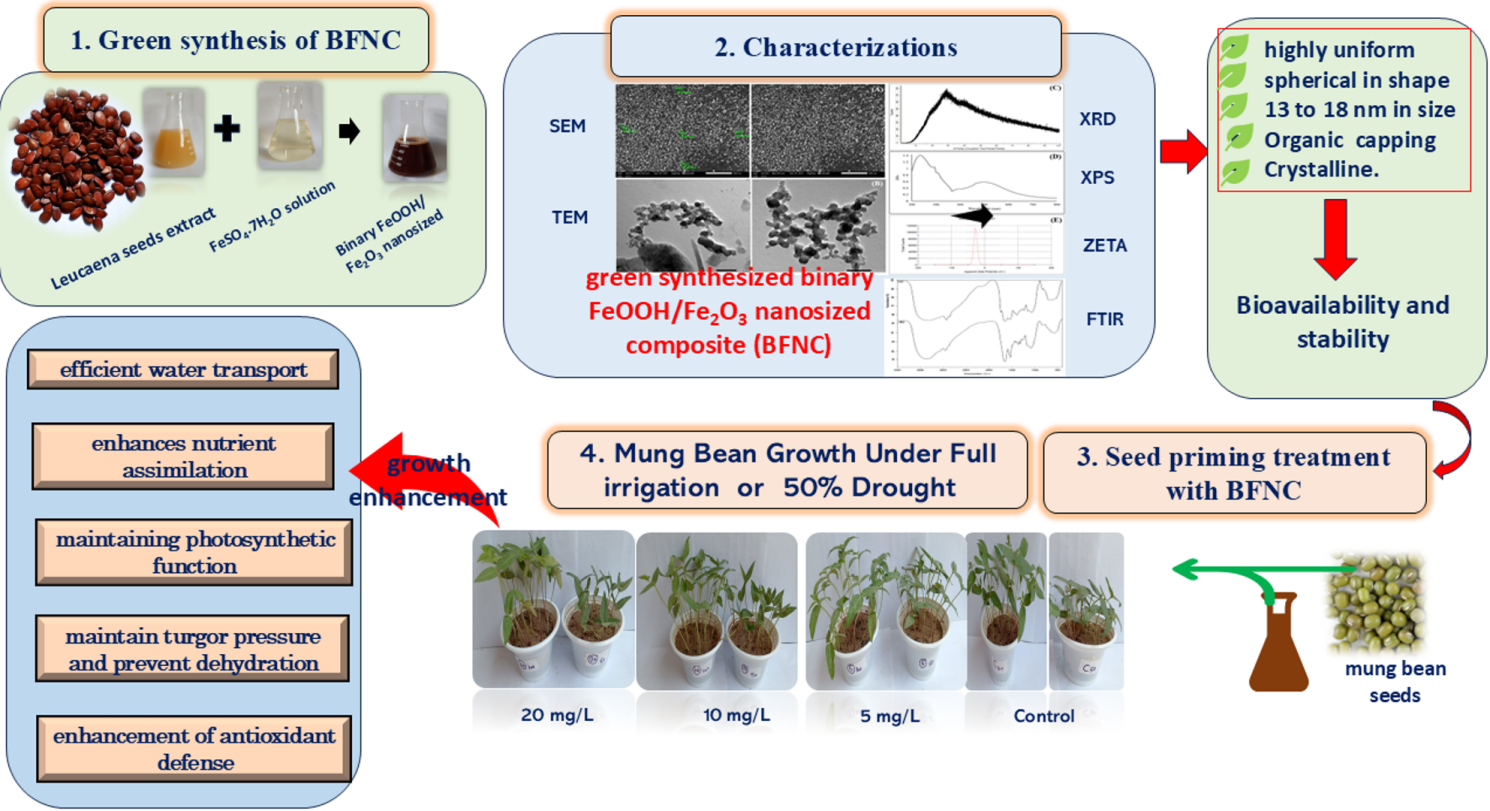

**Supplementary Information:**

The online version contains supplementary material available at 10.1186/s12870-026-08421-0.

## Introduction

Climate change is adversely affecting crop yields and is expected to continue doing so. This would consequently result in severe consequences for global food security [1, 2]. Drought is one of the most significant environmental stressors.Climate models predict significant increases in drought-induced yield loss for major crops, including wheat, maize, rice, and soybeans, without adaptation [3]. Drought stress impairs a plant’s ability to function properly, leading to a cascade of negative effects at physiological, biochemical, and molecular levels [4]. The negative impact of drought on plants occurs through reduced growth, damaged organelle function, disturbed nutrient uptake, degraded chlorophyll, and inhibited photosynthesis [5–7]. Drought stress severely affects plants by inducing oxidative stress and accelerating plant senescence [8].

There are several methods used to enhance water-use efficiency and crop resilience under drought conditions, such as the application of biochar as a soil amendments [9], administration of plant growth promoting bacteria [10], exogenous application of gibberellic acid [11], and pre-sowing treatment of seeds with nanoparticles (NPs) [12] .

The application of nanomaterials in agriculture is highly prospective due to their low concentration requirement and high efficacy .The use of nanoparticles in seed priming, known as nanopriming, offers a promising approach to enhance germination, seedling development, and crop productivity under drought stress [13]. NPs alleviate drought-induced stress in plants by regulating various physiological, biochemical, and molecular pathways, such as by improving water and nutrient uptake [14], boosting the activity of antioxidant enzymes [15], increasing the accumulation of osmolytes, which helps plants to cope with drought stress [16], and upregulating genes involved in drought tolerance [17]. Several types of NPs have been effectively utilized in seed priming to alleviate drought stress in plants, such as selenium NPs with tomato [18], copper NPs with wheat [19] and maize [20], titanium nanoparticles with dragonhead plants [21]and iron NPs with Sorghum [22].

Green synthesis of NPs is a more environmentally friendly approach, reducing the reliance on hazardous chemicals and physical methods, thereby providing significant ecological benefits [23].Green NPs exhibit substantially lower cytotoxicity and phytotoxicity compared to those synthesized using traditional chemical and physical methods, indicating that green NPs are harmless and can be broadly employed [24].

Green iron oxide nanoparticles (NPs) serve as a promising strategy to mitigate the adverse effects of drought on plants [25]. Dola et al. [26] reported that nano-iron oxide enhanced the growth, yield, and quality, and oil content of Glycine max under drought stress. Similarly, Naseem et al. [27] demonstrated that the application of green-synthesized iron oxide NPs improved growth, photosynthetic capacity, and antioxidant defense mechanisms of spinach plants during water scarcity. In addition, Bidabadi et al. [28] explored the efficiency of iron oxide NPs in enhancing antioxidant defenses in grapevines subjected to drought stress.

Generally, several phases are known for iron oxides due to their different oxidation states, comprising oxyhydroxide or hydroxide (FeOOH, Fe(OH)_2_ and Fe(OH)_3_) and iron oxide (Fe_3_O_4_, Fe_2_O_3,_ FeO) Remarkably, hematite (α-Fe_2_O_3_) is the most thermodynamically stable phase and other phases can be converted to α-Fe_2_O_3_ by pressure or heat treatment. However, FeOOH can be attained by the hydrolysis of iron salts. Iron oxide is easily prepared with diverse morphologies affording potential Fe²⁺ ↔ Fe³⁺ surface sites for redox reactions in an aqueous medium [29].

Strongly oxidizing hydroxyl radicals (.OH) produced by advanced oxidation processes (AOPs) are widely known for their ability to quickly and non-selectively oxidize the majority of organic compounds [30]. Among these, the Fenton reaction (Fe²⁺/H₂O₂) is appealing because of its high performance, ease of technology, and environmentally friendly reagents. The formation of insoluble ferric products is the primary limitation of Fenton systems, and hydroxyl radicals are produced in Fenton systems through the following reactions [31].

$$Fe^{2+}+H_2O_2 \rightarrow Fe^{3+}+^.\;OH+^-{OH}\;\;\;\;\;\;k=63M^{-1}\;s^{-1}$$

Mung bean (Vigna radiata L.) is a plant species belonging to the family Fabaceae. It is a multipurpose pulse crop with numerous uses [32]. Mung bean is a rich source of plant-based protein, containing 20–32% protein, making it a vital and affordable protein option, particularly in developing nations and for those following vegetarian or vegan diets [33, 34]. In addition, it contains a high amount of carbohydrates and dietary fiber [35]. Mung beans are rich in various essential vitamins, including vitamin B1, B2, B3, B6, and B9 [36]. This plant has been found to have therapeutic benefits, including antioxidant [37], anti-inflammatory [38], anti-diabetic [39], and blood pressure regulation properties [40].

Green iron oxide NPs have been shown to improve mung bean growth, with studies demonstrating increased germination percentage, shoot and root lengths, and seed vigor index when treated with Fe NPs synthesized from recycled kitchen waste [41] or biosynthesized from Opuntia stricta extract [42]. In addition, some studies have indicated the role of green iron oxide NPs in ameliorating abiotic stress in mung bean, such as arsenic toxicity [43] and UV-B stress [44].

Despite research on the effect of green iron oxide NPs on plants under drought stress and research on the effect of NPs on mung bean, to the best of our knowledge, the effect of green iron oxide NPs on mung bean under drought stress has not been investigated. Therefore, the current study aimed to investigate the role of green-synthesized binary iron oxides to mitigate the effect of drought stress on Vigna radiata (mung bean). In addition, we report the green synthesis of a binary FeOOH/Fe₂O₃ nanosized composite (BFNC) from the aqueous extract of Leucaena leucocephala seeds and freshly prepared ferrous sulfate via a Fenton’s-like experiment, in which the Leucaena leucocephala seed extract serves as an excellent oxidizing, capping, and stabilizing agent. However, as reported in most of the literature the green synthesis of iron based nanoparticles, utilized plant extract, as a bio reduction agent.

## Materials and methods

### Collection of seeds used in biosynthesis of iron oxide nano-particle

*Leucaena leucocephala* plant collected from house garden in El Beheira, Egypt. The seeds were washed with distilled water, air-dried, and ground to a powder using a blender. Fifty grams of seed powder was soaked in 1000 mL distilled water and left for 24 h under laboratory conditions to prepare the L. leucocephala extract. The extract was then filtered through Whatman No. 1 filter paper and stored in a refrigerator until further use.

### Green synthesis of a binary FeOOH/Fe_2_O_3_ nanosized composite (BFNC)

Iron sulphate was used as the metal precursor for the synthesis of the BFNC. A quantity of 0.83 g of iron sulphate was dissolved in 1000 mL of distilled water to prepare 3mM FeSO4.7H2O solution. This freshly prepared metal solution was mixed with the L. leucocephala seed extract in a volume ratio of 1:2 v/v. Bio-oxidation occured rapidly, as indicated by the development of a brick-red color. The mixture was then heated at 40 °C for one hour and kept at room temperature overnight. To harvest the synthesized BFNC product, the mixture was centrifuged at 5000 rpm for 20 min, The BFNC pellet was washed three times with distilled water, followed by a wash with 70% ethyl alcohol. After subsequent centrifugation, the resulting brick-red precipitate was dried at 30 °C for twelve hours. The BFNC was then subjected to various characterizations and used in the drought stress study.

### Physical and chemical characterization of the green synthesized binary FeOOH/Fe_2_O_3_ nanosized composite

Methods and instruments used to characterize the synthesized BFNC including X-ray photoelectron spectroscopy, Fourier transform infrared spectroscopy (FTIR), UV–Visible spectroscopy, energy-dispersive X-ray, scanning electron microscopy, transmission electron microscopy, X-ray diffraction, and zeta potential measurement, were described in the Supplementary Information (SI ).

### Investigation of the effect of green synthesized binary FeOOH/Fe_**2**_O_**3**_ nanosized composite on *Vigna radiata* under drought stress.

#### Seed nanopreiming

Mung bean seeds (Vigna radiata) were obtained from a local market in Al-Madina Al-Monoaurah, Saudia Arabia. Before germination, the seeds were surface-sterilized by immerseion in a 0.1% sodium hypochlorite solution for 3 min, followed by thorough rinsing with distilled water. The sterilized seeds were then soaked for 6 h in 100 mL of an aqueous BFNC solution at three different concentrations (5, 10, and 20 mg/L) [45].

#### Experimental conditions

The experiment was conducted in the garden of the Faculty of Science, Damanhour University, using a completely randomized design. Uniform seeds (15–20 seeds per pot) were sown in pots (14 cm in diameter and 10 cm in height) containing 1.0 kg of a sand-clay soil mixture in a 2:1 ratio. The experimental design consisted of the following treatments: T1 = control (without nanopriming); T2, T3, and T4 = seed priming with 5, 10, and 20 mg/L of the green-synthesized BFNC, respectively. All treatments were maintained at 100% (control) and 50% (drought stress) of the field water capacity. The experiment was performed under greenhouse conditions (20 ± 2 °C temperature, 75 ± 2% relative humidity, and a 14/10 h light/dark photoperiod). Three replicates per treatment were harvested for physiological and biochemical analyses.

#### Growth parameters

Growth parameters of mung bean shoots were measured, and leaf area was estimated using the equation by Milford et al. [46].

#### Determination of photosynthetic pigments

The photosynthetic pigments total chlorophyl (TCh ), chlorophyll a, (Cha ), chlorophyll b (Chb) were measured spectrophotometrically at 647 and 664 nm following N, N-dimethylformamide (DMF) method as described by Inskeep and Bloom [47]. The following formula and extinction coefficients were utilized to determine the the concentrations of the photosynthetic pigments:

- Chla = 12.70 A_665_ – 2.79 A_647_
- Chl.b = 20.70 A_647_ – 4.62 A_665_
- TCh = 17.90 A_647_ + 80.08 A_665_

#### Determination of soluble sugars and soluble protein

Protein content was quantified quantified using the modified Lowry method (Hartree 1972). Carbohydrate and protein contents of mung bean leaves were estimated as reported by Dubois et al. [48] and Hartree [49], respectively.

#### Determination of proline content

Free proline was extracted according to the method described by El-Sharkawi and Michel [50], its concentration was determined using the acid ninhydrin method described by Bates et al. [51].

#### Determination of phenolic content

The total phenolic content of the aqueous methanol extract of mung bean leaves was determined following the Folin-Ciocalteu method [52]. The reduction of the Folin-Ciocalteu reagent by phenolic compounds under alkaline conditions produces a blue color, which was measured at 760 nm using a spectrophotometer (Shimadzu UV-VIS2040 PC, Tokyo, Japan). The results were expressed as milligrams of gallic acid equivalent (GAE) per gram of dry mass [53].

#### Determination of catalase (CAT) and superoxide dismutase and glutathione reductase [GR] activities

The activities of catalase (CAT) and superoxide dismutase (SOD) were measured using commercial assay kits (Bio-Diagnostic and Research Reagents) according to Aebi [54] and Nishikimi et al. [55], respectively. Glutathione reductase activity was measured using the spectrophotometric assay described by Carlberg & Mannervik [56].

### Data analysis

All treatments were applied in three replicates. Data were subjected to analysis of variance (ANOVA), and mean differences were compared using the LSD test at a significance level of *p* < 0.05 [57]. Principal component analysis (PCA) was carried out using R software v.4.3.1 [58], correlogram was carried outusing Origin pro software 2026. Response Surface Methodology was employed using a Central Composite Design via Design-Expert software. Three-dimensional surface plots were generated to visualize the impact of the expected concentrations of BFNC (25–30 mg/L) on growth parameters and biochemical traits of *V. radiata.*

## Results

### Characterization of the green synthesized binary FeOOH/Fe_2_O_3_ nanosized composite

**Fig. 1:**
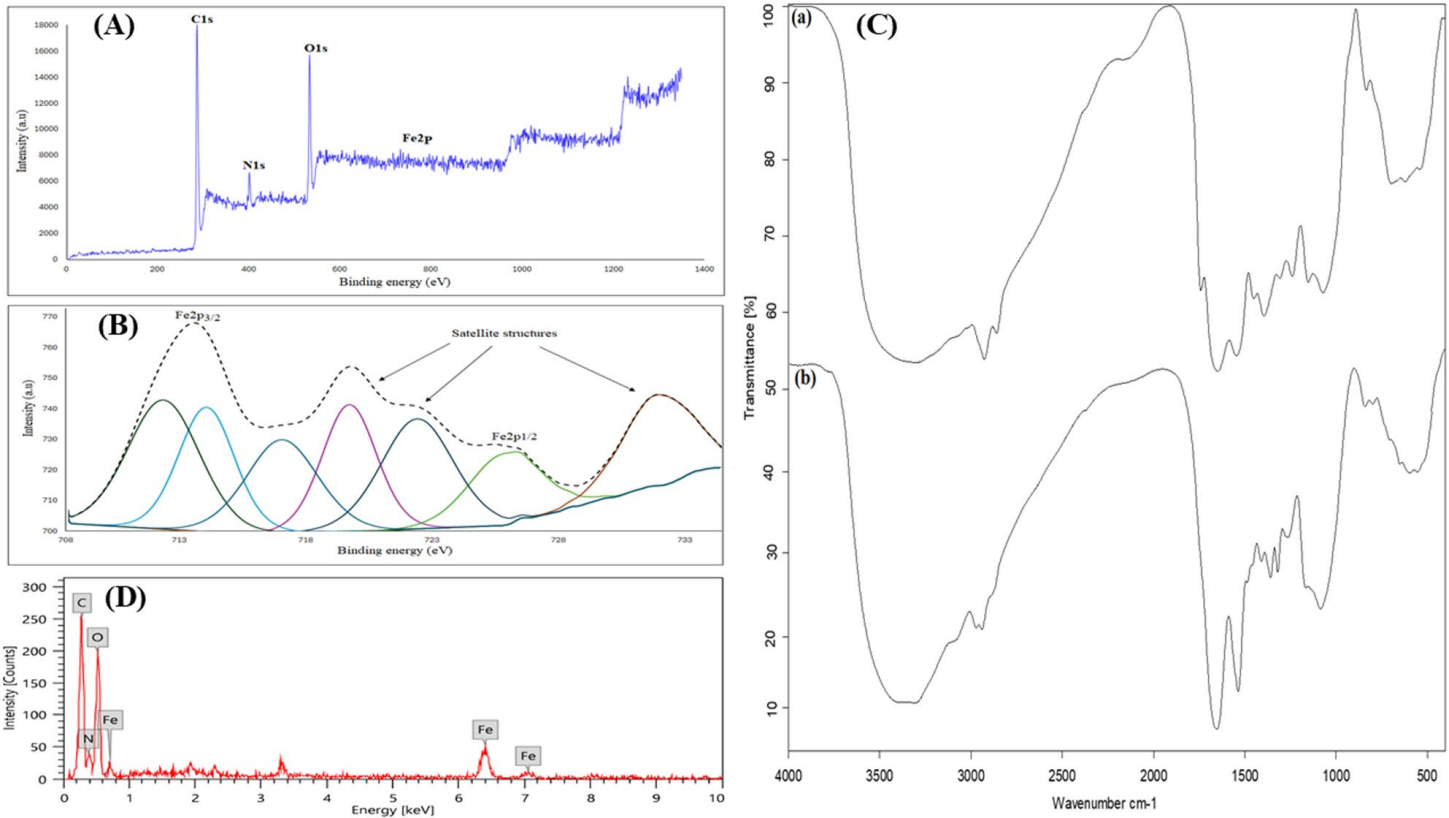
**A** Full scan XPS spectrum of binary FeOOH/Fe2O3 nanosized composite, **B** High resolution XPS spectrum of Fe2p, **C** Infrared spectra of (**a**) Leucaena leucocephala seeds extract, **b** binary iron oxides nanosized composite, and **D** EDS spectra of the prepared binary iron oxides nanocomposite

To evaluate the surface composition of the iron oxide nanosized composite, X-ray photoelectron spectroscopy (XPS) was used. The survey spectrum exhibited four peaks corresponding to the binding energy (B.E.) of C1s, N1s, O1s, and Fe2p at 286.14, 401.25, 533.13, and 716.36 eV, respectively (Fig. 1A). The high-resolution C1s spectrum (Figure S1) revealed three main peaks at 284.76, 285.83, and 287.58 eV. The deconvolution of the N1s peak (Figure S1) showed the presence of two nitrogen configurations at 399.68 and 401.04 eV. In addition, the O1s XPS spectrum (Figure S1) was deconvoluted into two primary peaks centered at ~ 531.22 and 532.51 eV. Furthermore, in the high-resolution Fe2p spectrum of the binary composite, multiple peaks are displayed, as shown in Fig. 1B.

The FTIR spectra of Leucaena leucocephala seeds and the synthesized binary FeOOH/Fe2O3 nanosized composite are presented in Fig. 1C.

The EDX results reveal the composition of elements present in the synthesized binary oxide nanosized composite (Fig. 1D). The detected elements include carbon (C), oxygen (O), nitrogen (N), and iron (Fe). Among these, carbon exhibited the highest weight% (wt%) of 44.90%.

**Fig. 2:**
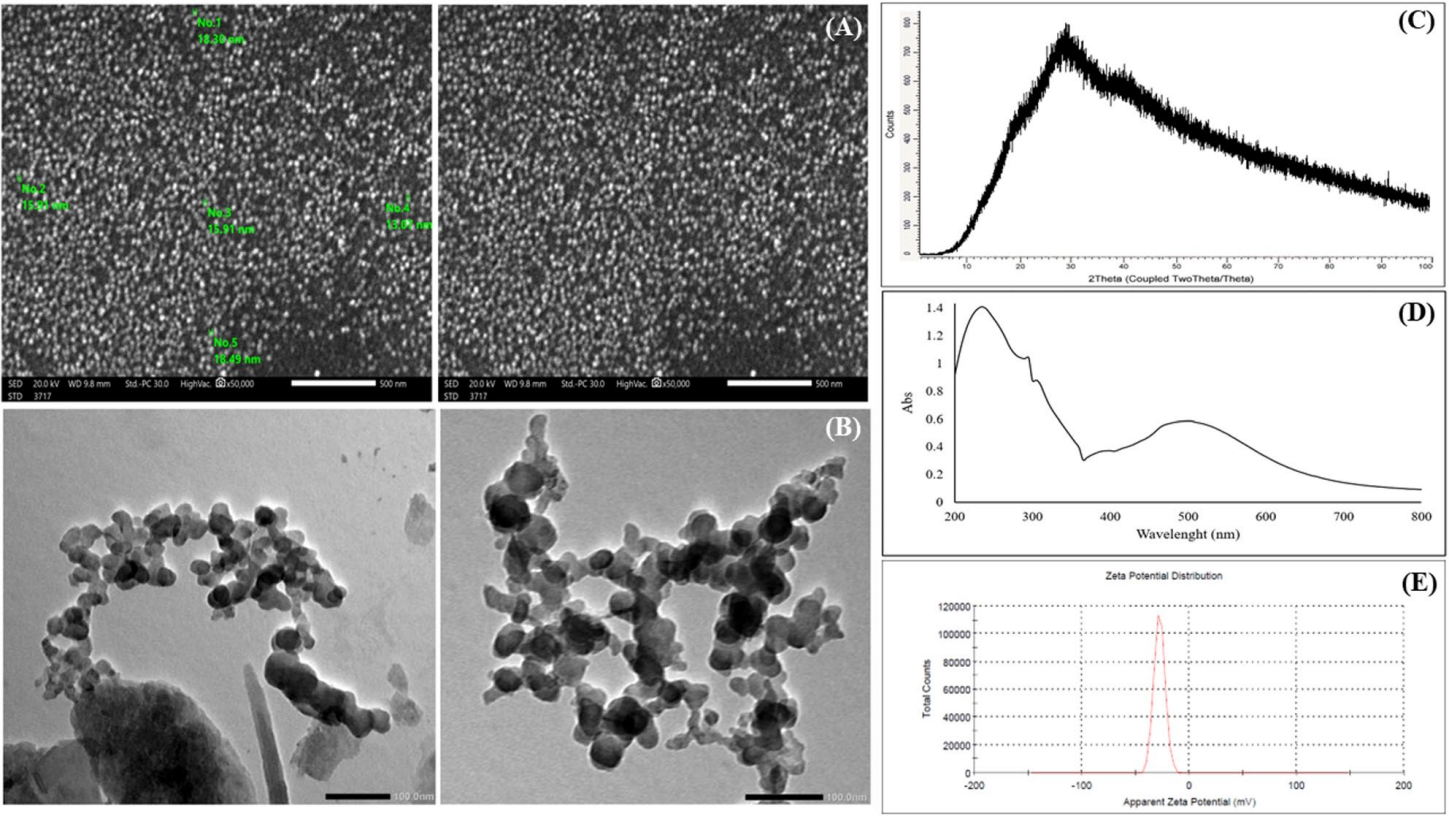
SEM images of the binary iron oxides nanosized composite (**A**), TEM images of binary iron oxides nanosized composite (**B**), XRD pattern of the binary iron oxides nanosized composite (**C**), UV–Vis absorption spectrum of binary iron oxides nanosized composite (**D**), and Zeta potential of binary iron oxides nanosized composite (**E**)

The average particle diameter of the prepared BFNC was determined from SEM images selected arbitrarily (Fig. 2A). The average diameter was 16.33 ± 1.97 nm, and the composite was composed of spherical NPs.

The transmission electron microscopy analysis confirms the sample’s nanoscale nature, with a relatively uniform and narrow size distribution for the individual nanoparticles, which have diameters ranging from 13.00 to 22.22 nm, as illustrated in Fig. 2B.

The XRD pattern of the synthesized binary iron oxides exhibited broad diffraction peaks (Fig. 2C), which are generally attributed to a particle size effect. The results revealed that the synthesized NPs were nanocrystalline in nature with a crystallite size of around 13.62 nm, as calculated by the Scherrer equation.

The electronic spectrum of the green synthesized binary oxides nanosized composite was recorded in water at the wavelength range of 200–800 nm. As illustrated in Fig. 2D, the electronic absorption spectrum exhibits absorption bands at 235 ,500 nm and a shoulder at 305 nm.

The negative charge on the binary nanoparticles was estimated from the zeta potential value (-27.8 mV) (Fig. 2E).

### Effect of the green synthesized binary iron oxides nanosized composite on growth parameters, of *Vigna radiata* under drought stress

**Fig. 3:**
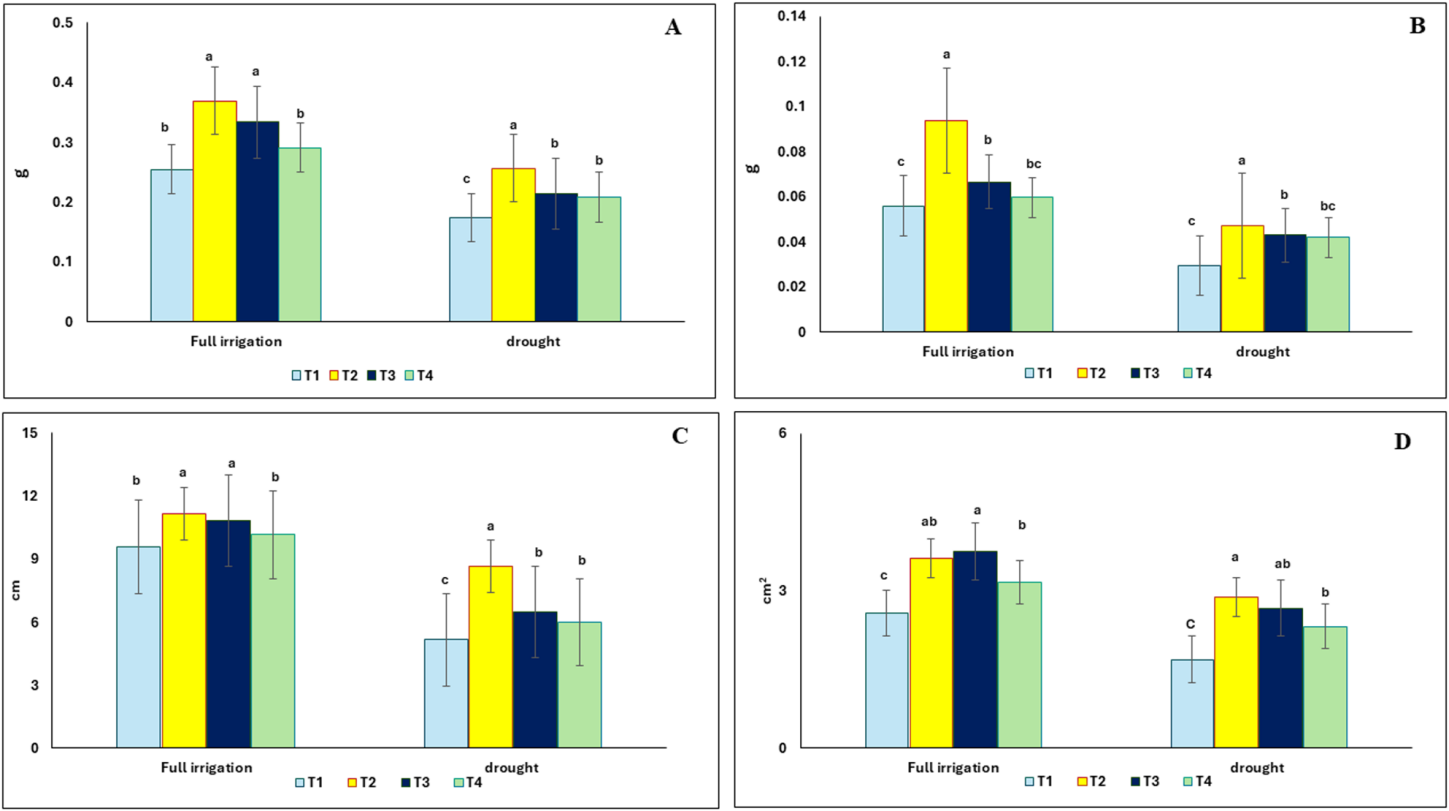
Effect of seed priming in aqueous binary iron oxides nanosized composite solution on shoot fresh weight (**A**), shoot dry weight (**B**), and shoot length (**C**), leaf area (**D**) of *V.radiata* under full irrigation or 50% drought stress. T1 = without nanopriming; T2, T3 and T4 = seed priming in 5, 10 and 20 mg/L iron oxide nano-particle, respectively. The values reported in the figure are means, Standard error bar is presented in the figures. Different letters on the bars are significantly different as evaluated by LSD Test at a significant level of *p* < 0.05

In general, the nanoparticles improved plant growth under both full irrigation and drought stress, with treatment T2 (5 mg/L) being the most effective, achieving the highest growth parameters. Drought stress significantly impacted the growth of V. radiata, reducing shoot fresh weight, shoot dry weight, shoot height, and leaf area by 32%, 47%, 46%, and 34%, respectively, compared to control plants (Fig. 3). However, seed priming in an aqueous BFNC solution at a concentration of 5 mg/L (T2) improved these parameters by 48%, 58%, 68%, and 67%, respectively, compared to drought-stressed plants.

### Effect of the green synthesized binary iron oxides nanosized composite on photosynthestic pigments, soluble sugars, soluble protein, proline and phenolics content of *Vigna radiata* under drought stress

**Fig. 4:**
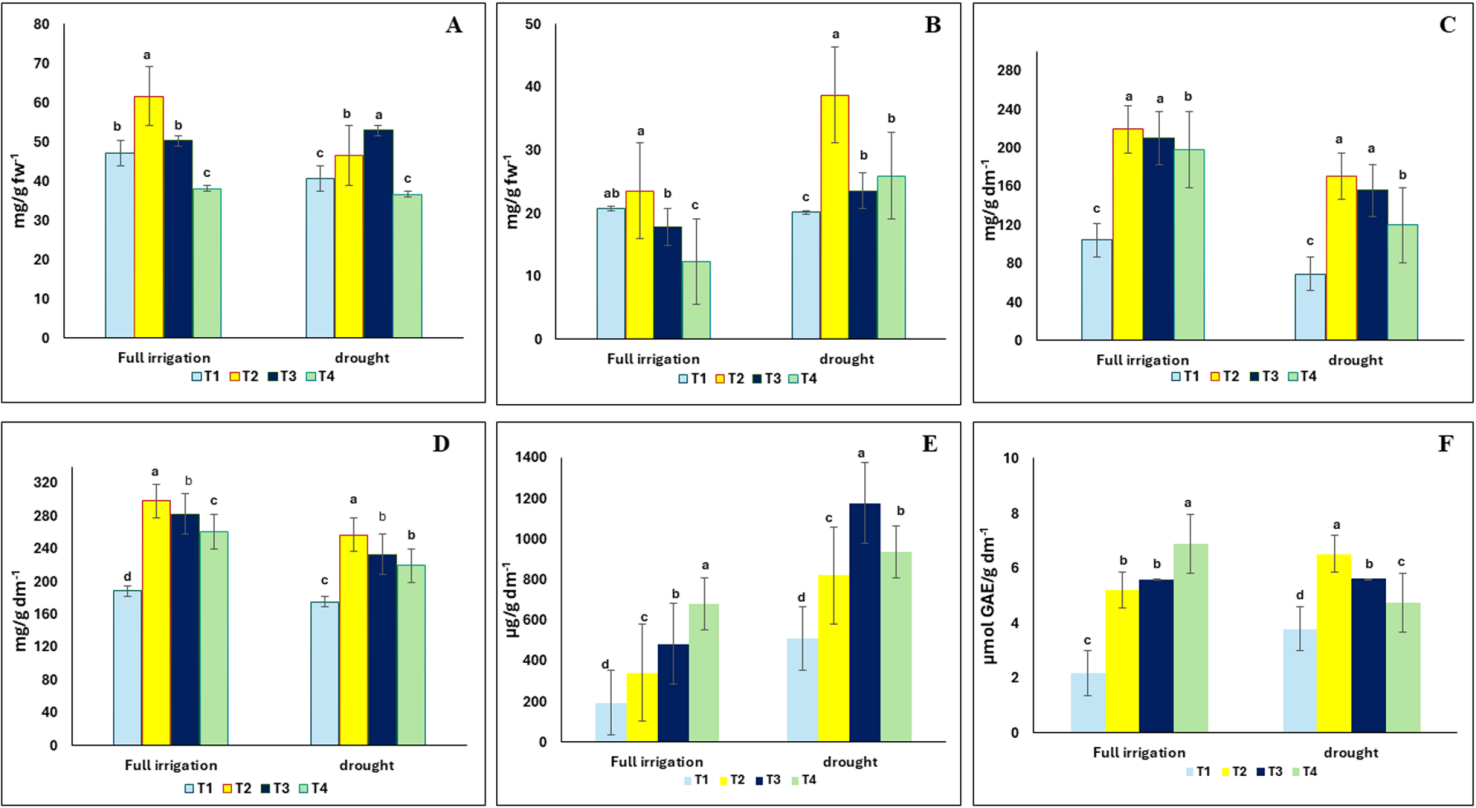
Effect of the green synthesized binary iron oxides nanosized composite on photosynthestic pigments, soluble sugars, soluble protein, proline and phenolics contents of *Vigna radiata* under drought stress. chlorophyll a (**A**), chlorophyll b (**B**), soluble sugars (**C**), soluble protein (**D**), proline (**E**), phenolics (**F**) of *V.radiata* under full irrigation or 50% drought stress. T1 = without nanopriming; T2, T3 and T4 = seed priming in 5, 10 and 20 mg/L iron oxide nano-particle, respectively. The values reported in the figure are means, Standard error bar is presented in the figures. Different letters on the bars are significantly different as evaluated by LSD Test at a significant level of *p* < 0.05

The effect of nanopriming and drought on the photosynthetic pigments of *V. radiata* is shown in Fig. 4A and B. Chlorophyll a decreased by 13% in drought-stressed plants compared to the control ones. However, nanopriming with an aqueous BFNC solution at a concentration of 10 mg/L substantially improved the content of chlorophyll a by 30% compared to non-treated control plants under drought stress. Regarding the content of chlorophyll b, nanopriming at a concentration of 5 mg/L resulted in a 92% increase under drought stress compared to the non-treated control. Furthermore, nanopriming (T2) improved chlorophyll a and b content under full irrigation conditions by 30% and 14%, respectively, compared to the control.

The effect of BFNC on the soluble sugars, soluble protein content is presented in Fig. 3C and D. Drought stress significantly reduced soluble sugars and soluble protein contents in *V. radiata*, whereas seed nanopriming with BFNC caused a significant increase in the content of soluble sugars and soluble protein by 146% and 47%, respectively, compared to the non-treated control plants. Likewise, the content of sugars and soluble protein increased significantly in response to nanopriming under full irrigation conditions. For example, treatment (T2) resulted in a two-fold and 1.5-fold increase in soluble carbohydrate and soluble protein contents, respectively.

The effect of nano-treatments on proline and phenolic content of mung bean is shown in Fig. 4E and F. Compared to the control, nano-treated plants showed a significant increase in proline and phenolic content, whether under full irrigation or drought stress conditions. For example, the proline content in plants treated with 5 mg/L of the BFNC solution increased by 148.84 µg/g dm^− 1^ compared to the control, while the phenol content increased by 3.02 µmol GAE/ gdm^− 1^ under drought stress.

### Effect of the green synthesized binary iron oxides nanosized composite on the activity of superoxide dismutase, catalase and glutathione reductase enzymes of Vigna radiata under drought stress

**Fig. 5:**
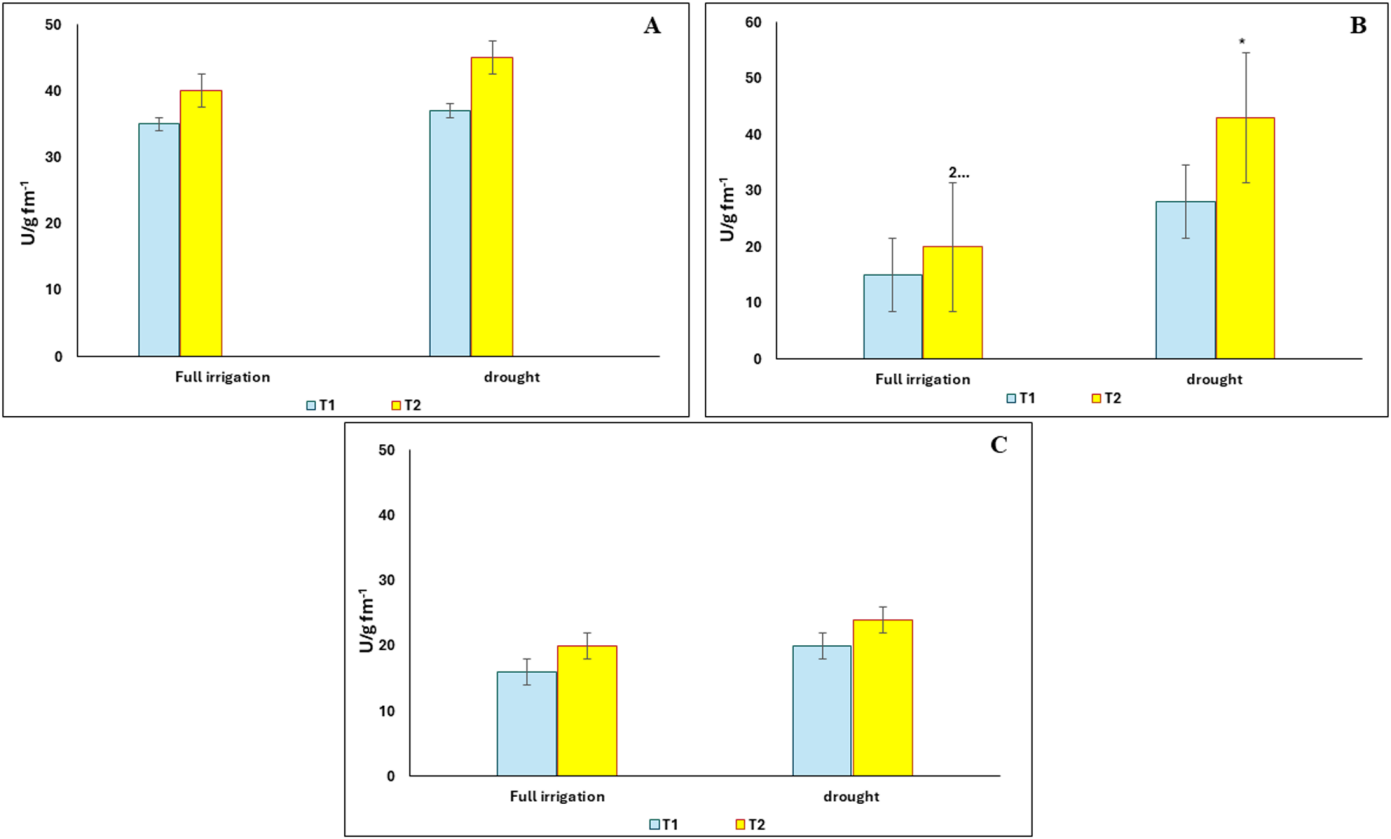
Effect of the green synthesized binary iron oxides nanosized composite on the activity of Superoxide dismutase (**A**), Catalase (**B**), glutathione reductase (**C**) enzymes of *V.radiata* under full irrigation or 50% drought stress. T1 = without nanopriming; T2, T3 and T4 = seed priming in 5, 10 and 20 mg/L iron oxide nano-particle, respectively. The values reported in the figure are means, Standard error bar is presented in the figures. * =significant difference at *p* < 0.05

As shown in Fig. 5, nano-treatment under stress conditions significantly enhanced the activity of catalase enzyme under drought stress, whereas the increases in the activity of superoxide dismutase and glutathione reductase enzymes were not statistically significant.

### Principal component and correlation analysis

**Fig. 6:**
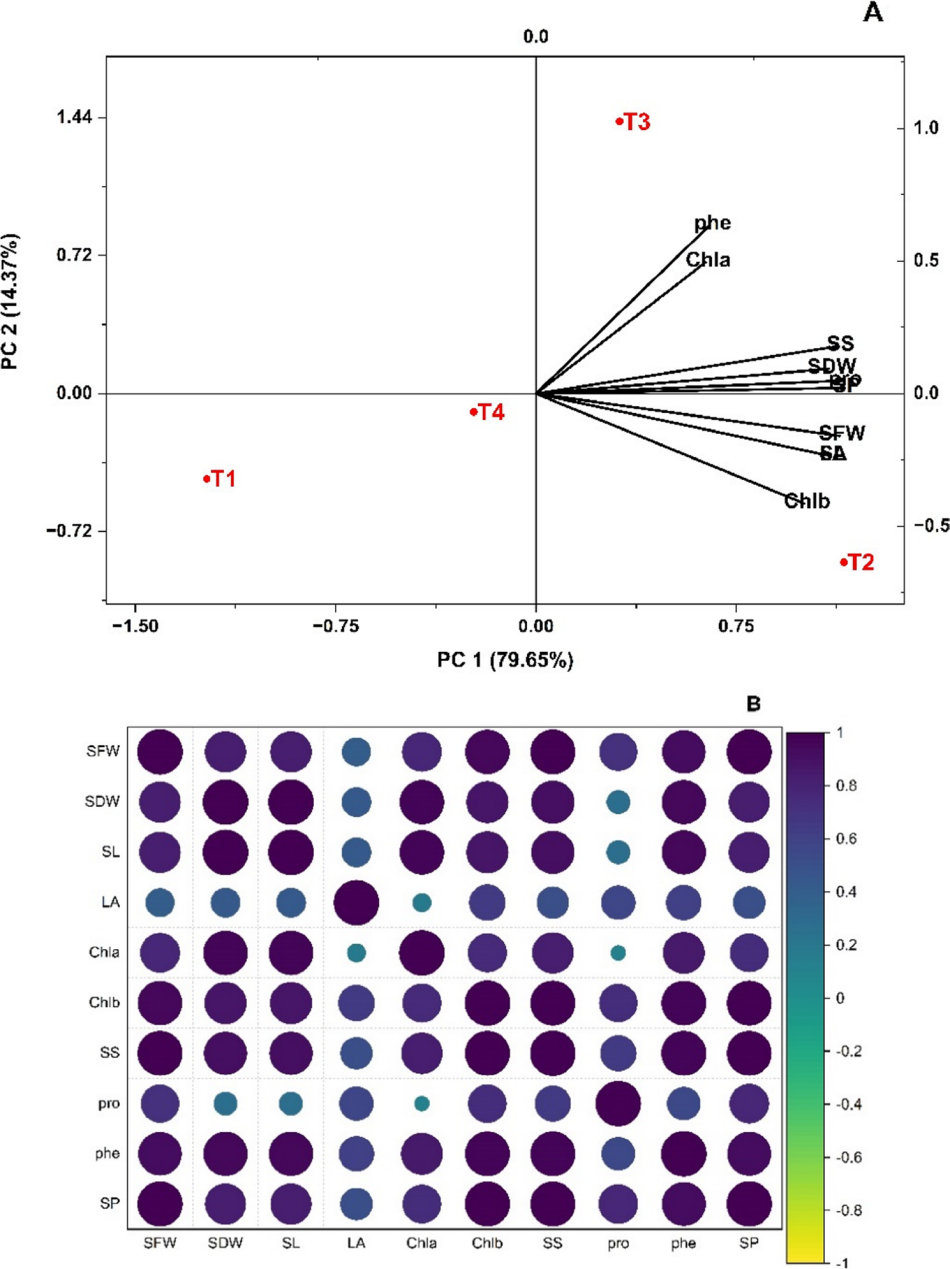
(**A**) Biplot for the principal component analysis, (**B**) Correlogram based on the correlation coefficients between growth parameters and biochemical traits of *V.radiata* in response to priming with binary iron oxides nanosized composite under drought stress. T1 = without nanopriming; T2, T3 and T4 = seed priming in 5, 10 and 20 mg/L, SFW= shoot fresh weight, SDW= shoot dry weight SL= shoot lenghth, LA= leaf area, Cha= chlorophyll a, Chb= chlorophyll b, SS= soluble sugars, Pro= proline, Phe= phenolics Sp= soluble protein

The PCA of growth parameters and biochemical traits of *V. radiata* in response to nanopriming with BFNC under drought stress is presented in Fig. 6A. The first two principal components, PCA1 and PCA2, accounted for 79.65% and 14.37% of the total variation, respectively, collectively explaining 94.02% of the data variability. The PCA revealed that *V. radiata* plants treated with 5 mg/L BFNC (T2) differed distinctly from the control and other nano-treated plants in terms of growth parameters and biochemical traits. Correlograms based on the correlation coefficients between growth parameters and biochemical traits of *V.radiata* in response to priming with binary iron oxides nanosized composite under drought stress shown in Fig. 6B. It is apparent from the correlogram that all variavles are positively correlated with each other, with shoot fresh weight, shoot dry weight, soluble sugars and soluble protein are strongly correlated.

### Factorial analysis

**Fig. 7:**
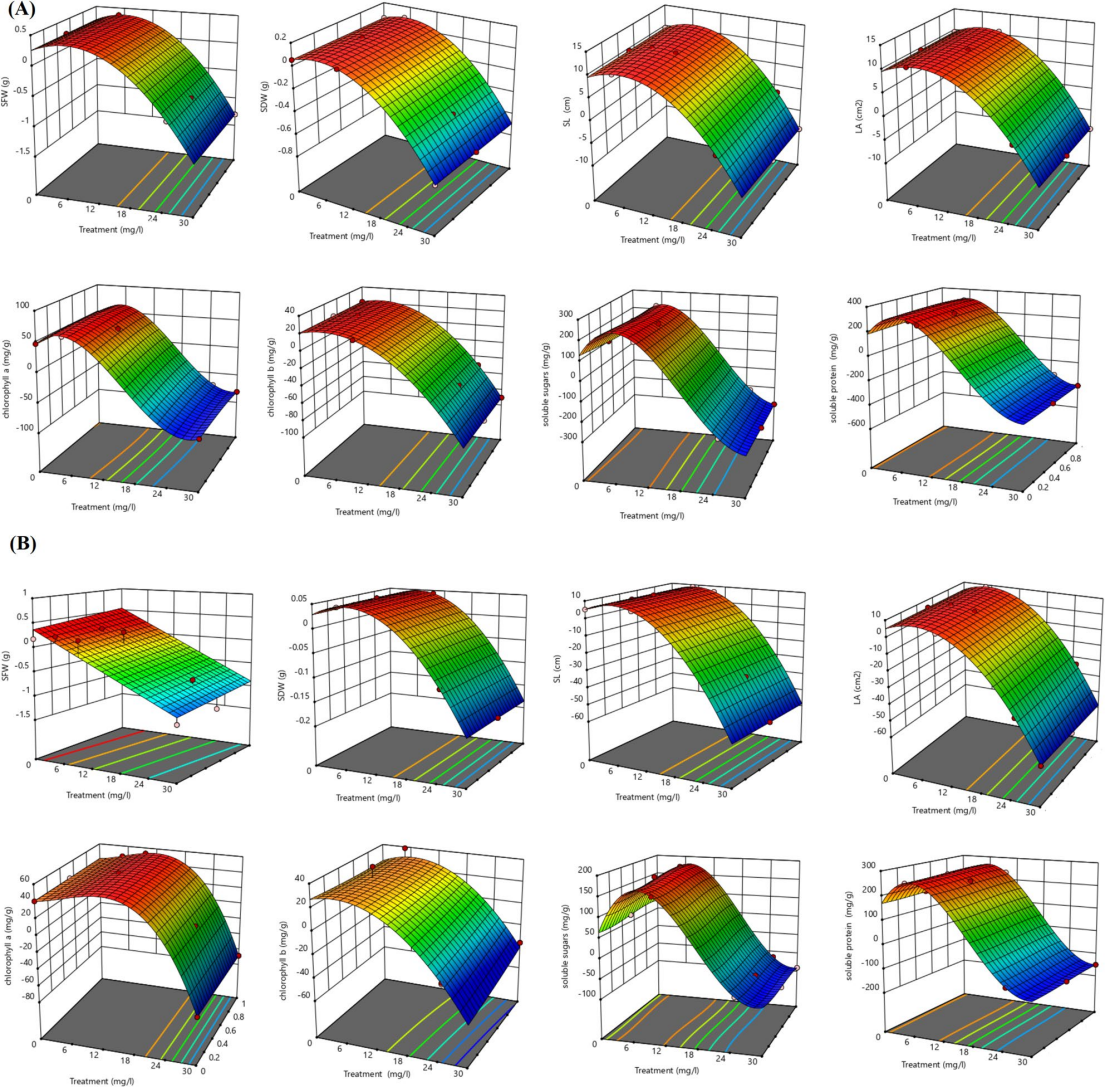
Response surface plot demonstrate the effect of estimated concentrations of BFNC (25, 30 mg/L) on shoot fresh weight, shoot dry weight, shoot length, leaf area, chlorophyll a, chlorophyll b, soluble sugars and soluble protein of *V.radiata* under control (**A**) or drought stress (**B**)

Figure 7 shows the three-dimensional response surface plot of growth parameters and biochemical traits of V. radiata under elevated concentrations of BFNC (25 and 30 mg/L). Increasing the concentration of BFNC resulted in a significant decrease in shoot fresh weight, shoot dry weight, shoot length, leaf area, chlorophyll a, chlorophyll b, soluble sugars, and soluble protein.

## Discussion

The XPS survey spectrum of the iron oxide nanosized composite exhibited four peaks corresponding to the binding energy (B.E.) of C1s, N1s, O1s, and Fe2p at 286.14, 401.25, 533.13, and 716.36 eV, respectively. The high-resolution C1s spectrum (Figure S1) revealed three main peaks at 284.76, 285.83, and 287.58 eV. The first peak arose from the C–(C, H), C = C (sp²-bonded carbons), and C–C (sp³-bonded carbons) moieties. The second peak corresponds to C–(N, O) moieties, and the third peak corresponds to C = O and O–C = O moieties, suggesting the presence of organic contaminants from Leucaena leucocephala seeds, which are composed of tannins, organic acids, oils, and amino acids.

Furthermore, the deconvolution of the N1s peaks showed the presence of two nitrogen configurations at 399.68 and 401.04 eV, which can be assigned to C = N, NH₂, the azo group (N = N), and pyrrole (NH), respectively. In addition, the O1s XPS spectrum was deconvoluted into two primary peaks centered at ~ 531.22 and 532.51 eV. Moreover, in the high-resolution spectrum of the binary composite, the Fe2p region displays multiple peaks, as shown in Fig. 1B. The main Fe2p₃/₂ and Fe2p₁/₂ peaks at ~ 712.28 and ~ 726.18 eV, respectively, with a peak separation (ΔB.E. = 13.9 eV) along with well-defined multiple shake-up satellite structures at 717.08, 719.78, 722.48, and 732.08 eV, strongly suggest the presence of the Fe³⁺ oxidation state, which is characteristic of iron oxides such as Fe₂O₃ and/or iron oxyhydroxide (FeOOH) [59]. The Fe³⁺ species exhibit higher binding energies than Fe²⁺ species (which typically appear at ~ 709–710 eV) due to their higher oxidation state, which is associated with an enhanced effective nuclear charge. In the Fe³⁺ oxidation state, the loss of three electrons raises the binding energy in XPS spectra by strengthening the attraction between the nucleus and the core-level electrons [60].

The presence of a broad O1s peak at ~ 531.22 eV is attributed to the Fe–O bond (O²⁻) in hematite (Fe₂O₃), while the peak centered at ~ 532.51 eV corresponds to the hydroxyl groups (OH⁻) in FeOOH [61]. These findings suggest the coexistence of FeOOH and Fe₂O₃ phases on the surface of the prepared sample as a BFNC. We can infer from the data above that the Fe/Leucaena leucocephala seed extract system is a Fenton’s-like experiment in which H₂O₂ is not included. The reaction would begin with the transfer of an electron from one of the rich functionalities found in the seed extract, such as –COOH, –OH, –NH₂, or –SH, to the water-dissolved oxygen to form the radical oxygen (O₂•⁻) in situ. This would either oxidize the ferrous ion to its brown ferric Fe³⁺, as shown in Eq. (1), or dismutate to H₂O₂ (Eq. (3)), and the formed H₂O₂ would then react with Fe²⁺ to produce •OH (Eq. 4). Iron can therefore undergo oxidation [62–64].

1 $$Fe^{2+}+\;O^2\rightarrow\;O^{2\bullet {-}}+\;Fe^{3+}$$

2 $$L. leucocephala seeds + O^2 \rightarrow O^{2\bullet{-}}$$

3 $$O^{2\bullet{-}} + O^{2\bullet{-}} + 2H^+ \rightarrow H_2O_2$$

4 $$Fe^{2+} + H_2O_2 \rightarrow Fe^{3+} + OH^\bullet + OH^{-}$$

5 $$Fe^{3+} + 3OH^- \rightarrow Fe(OH)_3$$

6 $$Fe(OH)_3 \rightarrow FeOOH+{H}_2{O}$$

7 $$2FeOOH \rightarrow \alpha-Fe_2O_3+{H}_2{O}$$

The FTIR spectrum of Leucaena leucocephala seeds exhibited several characteristic absorption bands assigned to various functional groups, including hydroxyl, carboxyl, amide, and carbonyl groups. These functional groups align with the primary components of *L. leucocephala* seeds, which are mostly composed of tannins, organic acids, oils, and the amino acid mimosine [65, 66]. The broad band at 3305 cm⁻¹ corresponds to stretching hydroxyl (O–H) groups present in the plant material. The absorption peaks observed at 2926 and 2859 cm⁻¹ are allocated to the υ(C–H) of hydrocarbon chains [67]. Peaks at 1741 and 1648 cm⁻¹ can be attributed to the asymmetric and symmetric stretching modes of carboxyl (–COOH) and/or –COO⁻ groups of amino acids. Furthermore, the sharp peak at 1648 cm⁻¹ may also be related to the stretching and bending vibrations of adsorbed water molecules (H₂O) or structural hydroxyl groups. The peaks at 1546 and 1395 cm⁻¹ are attributed to the C = O stretching vibration of the amide (–NH–C = O) bond and N–H moiety of primary amines bending and methylene (–CH₂), respectively [68]. The peak appearing at 1240 cm⁻¹ is most likely associated with the C–O stretching of polyols present in glucans and flavones [69]. The absorption bands at 1153 and 1072 cm⁻¹ are ascribed to the C–O–H stretching vibrations originating from mannose structures [70]. Bands of organic functional groups obtained from extracts may overlap with bands of metal–oxygen vibrations [71]. The FTIR spectrum of the binary composite revealed the existence of a new peak at 3379 cm⁻¹, which is ascribed to hydroxyl stretching, indicating the capping efficacy of L. leucocephala extract. The new peaks at 588, 788, and 831 cm⁻¹ can be assigned to the bending vibration of Fe₂O₃ and Fe–O–H in the (FeOOH/Fe₂O₃) nanocomposite, respectively [72]. Additionally, all the characteristic peaks identified in the plant powder were shifted, confirming the successful formation of the capped binary iron oxide [73].

The elemental composition of the synthesized binary oxide nanosized composite was determined by energy-dispersive X-ray analysis. The detected elements were carbon, oxygen, nitrogen, and iron. The highest weight% of carbon (wt%) was 44.90%. This result revealed that the plant extract used in the synthesis of the binary oxide nanosized composite is primarily composed of sugars, which accounts for the sample’s high carbon content. The coexistence of oxygen and carbon suggests that the plant material utilized in the synthesis is predominantly polysaccharidic in nature.

The morphology of the binary iron oxide nanosized composite was characterized using a scanning electron microscope (SEM). The synthesized binary composite exhibited a well-separated spherical nanoparticle morphology. The average diameter of the particles, assessed from SEM pictures by arbitrary selection, ranged from 13 to 18 nm, and interconnection between particles existed to a lesser extent. The propensity for spherical particle formation may be interrelated to the dispersion stability of particles in the reaction solution or the precipitation mechanism of the particles [74]. In addition, the BFNC effectively suppresses the aggregation and interconnection of the particles and strengthens the structural stability [75].

To analyze the microstructure of the FeOOH/Fe₂O₃ nanocomposites, transmission electron microscopy was performed. TEM analysis clearly confirms the nanoscale nature of FeOOH/Fe₂O₃, providing key morphological and structural information that is in line with the SEM and XRD data. The micrograph shows that the nanosized composite has an approximately spherical to irregular morphology, with substantial aggregation. The observed aggregation, or agglomeration, is a common phenomenon in fine nanopowders due to the high surface energy and attraction forces between particles [76].

The XRD pattern of the synthesized binary iron oxides exhibited diffraction peaks at 2θ values around 18°, 28°, and 39°, which could be accounted for by the crystal planes (110) of FeOOH and (104), (113) planes of Fe₂O₃, respectively. These are in good agreement with the standard JCPDS card No. 01-0136 and are attributed to Fe + 3O(OH)/Fe2O3.H2O Lepidocrocite, which clearly show that the the synthesized binary iron oxides were successfully formed. The peak broadening in the XRD pattern may be attributed to the coexistence of mixed iron oxide nanoparticles (FeOOH and Fe₂O₃), which leads to overlapping reflections and less distinct diffraction peaks that cannot be clearly distinguished ; this may be due to their nearly similar crystal structures [77, 78].

The average crystallite size was 13.62 nm, as calculated by the Scherrer equation; this small size may be attributed to the stabilizing effect of the plant extract’s biomolecules, which prevent excessive grain growth. Overall, the XRD results suggest the formation of a mixed-phase iron oxide system (primarily FeOOH with Fe₂O₃) coated with organic residues from the plant extract, which acted as stabilizing and natural capping agents throughout the biosynthesis process. Thus, the XRD analyses confirmed the capability of the adopted method to preserve the FeOOH/Fe₂O₃ phase through the green synthesis process of the Fe/ Leucaena leucocephala seeds extract system.

The electronic absorption spectrum of the green-synthesized binary oxide nanosized composite exhibits a high-intensity absorption band observed at 235 nm, which is assigned to π–π∗ transitions associated with aromatic rings and conjugated systems present in polyphenols, flavonoids, and other biomolecules derived from the capping plant extract, which are adsorbed onto the surface of the nanoparticles [79] and contribute to their optical properties and surface stabilization. The broad absorption band observed in the visible region at λ = 500 nm can be ascribed to d-d transitions in the binary oxides [27]. However, as reported in earlier studies [80], both Fe₂O₃ and FeOOH display electronic absorption bands around λ = 500 nm.

The small shoulder at λ = 305 nm is due to a charge transfer (CT) of non-bonded electrons from O(2p) to Fe (III), indicating overlapping electronic transitions from different phases [81]. Thus, the successful production of the FeOOH/Fe₂O₃ nanocomposite using the plant extract is confirmed by the UV-Vis absorption spectrum.

The stability of the synthesized binary oxide nanosized composites was confirmed by the Zeta potential value. The particles demonstrated good stability, as the zeta potential was − 27.8 mV, implying their stability in the environments in which they are used [82].

Drought is the most damaging abiotic stress in the agricultural sector and is likely to affect more than 50% of agricultural land by 2050 as a result of climate change [83, 84]. Drought stress leads to significant reductions in plant growth, productivity, and yield, causing a widespread food crisis [85]. The present study revealed that drought exerted negative influences on mung bean growth parameters, photosynthetic pigments, soluble sugars, and protein contents. However, nanopriming of mung bean seeds with the green binary iron oxide nanosized composite at a concentration of 5 mg/L resulted in a significant amelioration of the aforementioned attributes. The present findings appear to align with other studies indicating that green-synthesized iron oxides exhibit environmental compatibility and effectiveness in promoting plant growth under drought [28, 86, 87]. The positive effect of iron oxide NPs on drought-stressed mung bean plants has also been observed in several other crops, such as moringa [88], barley [89], soybean [26], wheat [17], garden pea [90], and maize [91], through different methods of application, including foliar spray, seed priming, and soil amendment.

There are several mechanisms that explain the significant potential of iron oxides NPs in enhancing plant growth and resilience under drought stress. Iron oxides NPs improve water absorption by seeds, leading to a significant increase in germination rates and seedling establishment under drought conditions [92]. This is achieved by modulating aquaporin activity, which facilitates more efficient water transport within plant tissues, resulting in higher leaf relative water content and improved water potential in plants under drought stress [93]. In line with this context, Faisal et al. [94] reported that the higher surface reactivity of nanoparticles enables them to create pores in roots, thereby facilitating increased nutrient and water movement within plants. In addition, iron oxides NPs act as a nutrient delivery system, ensuring that essential micronutrients like iron are readily available to plant roots [95]. This delivery enhances nutrient assimilation, which supports healthier plant growth and higher yields, even under stress conditions [96]. Furthermore, iron oxides NPs contribute to the stabilization of chlorophyll and other photosynthetic pigments, thereby maintaining photosynthetic function under drought conditions [97]. In line with this context, the current study’s results showed that nanopriming significantly induced chlorophyll a and b content in mung bean plants under drought stress.

One of the mechanisms that explains the improvement in plant growth when treated with NPs under drought stress is the ability of NPs to enhance osmolyte accumulation, such as proline and soluble sugars [98]. In the current study, plants treated with iron oxide NPs showed a significant increase in soluble sugars and proline content relative to untreated plants under drought conditions. Soluble sugars play a pivotal role in mitigating drought stress in plants by preventing water loss and maintaining cell structure integrity under drought conditions through osmotic regulation and water retention [90], by providing energy [99], and by exhibiting antioxidant properties [100].

Similarly, proline plays a crucial role in plant drought tolerance, serving multiple protective functions. As an osmolyte, proline helps cells retain water, maintain turgor pressure, and prevent dehydration in plants subjected to drought [101]. Proline mitigates oxidative stress by scavenging free radicals and reducing reactive oxygen species (ROS) levels, thereby maintaining cellular redox balance and protecting membrane and protein integrity under drought conditions [102, 103]. It also stabilizes crucial enzymes and protein complexes, including RUBISCO and mitochondrial electron transport components, that are vital for maintaining plant metabolism during stress conditions [104]. Furthermore, proline metabolism helps regulate the energy balance between chloroplasts and mitochondria, thereby supporting plant survival and growth both during and after drought stress [105].

As a metabolic adaptation, the accumulated soluble sugars interact with amino acid metabolism (e.g., proline), which together enhance the plant’s ability to mitigate drought stress. In line with this context, the results of the current research showed a significant increase in the content of phenolic compounds in treated plants compared to control plants. The application of iron nanoparticles (Fe NPs) under drought stress has been reported to stimulate the accumulation of phenolic compounds in some plants, for example, rosemary [106] and lemon balm [100]. Fe NPs can boost the activity of PAL and other enzymes in the phenylpropanoid pathway, leading to enhanced production of phenolic compounds [107]. The accumulation of phenolic compounds in response to Fe NP treatment under drought stress leads to the enhancement of antioxidant defense [108], protection of the photosynthetic apparatus [109], and osmotic adjustment and cellular integrity [110].

Notably, this shift towards non-enzymatic antioxidant mechanisms was further supported by the lack of significant changes in SOD and GR activities in mung bean plants treated with NPs under drought stress, suggesting that NP treatment primarily activated non-enzymatic antioxidant and osmoprotective mechanisms rather than enzymatic antioxidant defenses. These responses may have maintained cellular redox homeostasis, reducing the need for SOD and GR upregulation. Consequently, this resulted in decreased cellular damage and improved preservation of cellular structures like mesophyll cells, which are essential for photosynthesis.

Factorial analysis from this study indicates that higher concentrations of the BFNC (25 and 30 mg/L) resulted in a significant decrease in growth parameters and biochemical attributes of *V. radiata*. Therefore, the application of the BFNC should be at a low concentration (5 mg/L) to avoid toxicity. This finding is consistent with previous studies, where Ingle et al. [111] and Chandran Prasanna Ramachandran et al. [112] reported that nanocomposites and NPs can promote plant growth at optimal or low concentrations, but higher concentrations often result in toxicity and reduced growth. Reduced plant growth at higher nanocomposite applications can be attributed to oxidative stress and ROS generation [113], genotoxicity and DNA damage [114], disruption of metabolic and cellular processes [115], and physical damage to cells and organelles [116].

The results of the current study show that the BFNC has a positive effect on mung bean plants under both full irrigation and drought stress, particularly at a concentration of 5 mg/L. This positive effect at a low concentration may be attributed to the advantages of a binary FeOOH/Fe₂O₃ system that are not achievable with single-phase iron oxides, such as interfacial synergism (heterojunction formation). Unlike single-phase oxides, the binary interface creates a junction that facilitates faster electron transfer and prevents charge recombination [117, 118], making the composite more electrochemically active and efficient in triggering plant metabolic pathways. In addition, single-phase oxides are often either too stable (unavailable to the plant) or too reactive (toxic). Our binary composite, with its small crystallite size (13.62 nm), provides a controlled and sustained release of iron, acting as a high-performance nanopriming agent.Some studies have highlighted the role of nanosized composites in enhancing plant growth under stress conditions. For example, Ungson et al. [119] reported that graphene–Cu nanocomposites induced tomato tolerance to *Fusarium oxysporum* and increased antioxidant activity, while Abdullah et al. [120] indicated that an Iron/Copper/Phosphate nanocomposite enhanced growth parameters and mitigated drought stress in wheat. In addition, the application of biochar-based metal oxide nanocomposites decreased salt toxicity and improved safflower growth [121]. Similarly, a recent study by Fayad et al. [122] reported that biochar and cerium oxide nanoparticles mitigated nickel and drought stress in chickpea.

Additionally, the advanced techniques employed in the current study to characterize the green synthesized revealed that the green synthesized binary FeOOH/Fe_2_O_3_ nanosized composite were highly uniform, spherical in shape, and predominantly ranged between 13 and 18 nm nm in size (Fig. 2A and b), which is critical for predictable interactions with plant cells and the consistent delivery of micronutrients.The uniform nanoparticle size enhances bioavailability and facilitates efficient uptake by plant tissues [123]. In line with this context, the small size of green synthesized BCNP allow them to penetrate plant tissues and interact with biological systems at the molecular level [124].FTIR spectroscopy confirmed the presence of O–H, C–H, –COOH, and C = O functional groups (Fig. 1C), indicating that organic residues from *L. leucocephala* seeds act as natural capping agents. This organic coating enhances nanoparticle stability, facilitates interactions with biological membranes, and improves bioavailability [123], thereby promoting compatibility with plant systems [125]. XRD analysis of the green-synthesized BFNC revealed a crystalline structure. The nanoscale size and crystallinity confer high surface energy and reactivity, properties that are critical for integration into plant metabolic pathways during adaptation to abiotic stress [125]. Zeta potential measurements further confirmed the stability of the synthesized binary oxide nanosized composites. Collectively, these distinct physicochemical characteristics suggest that green-synthesized BFNC may effectively activate stress-responsive pathways under drought conditions.

## Limitations of the study and o future research directions

A key limitation of this study is the potential phytotoxicity of the green-synthesized binary FeOOH/Fe₂O₃ nanosized composites (BFNC) at elevated concentrations. While the effects of 25–30 mg/L BFNC on growth parameters and biochemical traits of V. radiata were visualized using three-dimensional surface plots, further experiments at higher concentrations are necessary to assess toxicity risks. Another limitation is the environmental fate and risks of BFNC are not yet fully understood. Future research should investigate uptake mechanisms, long-term stability in plant tissues, translocation across trophic levels, and persistence within agroecosystems. Addressing these gaps is critical for the safe and sustainable integration of nanotechnology into agriculture, particularly with the increasing challenges of climate change.

## Conclusion

The combination of L. leucocephala seed extract and ferrous ions induces the Fenton-like reaction and leads to the oxidation of iron. Characterization by FTIR, UV-Vis, EDX, XPS, XRD, SEM, TEM, and zeta potential confirmed the formation of a binary FeOOH/Fe₂O₃ nanocomposite. Nanopriming of mung bean seeds in an aqueous solution (5 mg/L) of the green-synthesized binary FeOOH/Fe₂O₃ nanocomposite resulted in improved shoot dry weight (58%), shoot height (68%), leaf area (67%), and protein content (47%). It also increased the activity of the non-enzymatic antioxidant defense system (proline 61%; phenolic content 74%). According to these results, the application of the green-synthesized binary FeOOH/Fe₂O₃ nanocomposite offers a promising, cost-effective, and eco-friendly practice to mitigate the negative impacts of drought stress on crops. This aligns with the Sustainable Development Goals (SDGs), which aim for climate action and maintaining food security.

## Supplementary Information

Supplementary Material 1.

Supplementary Material 2.

## Data Availability

All data generated or analyzed during this study are included in the current article.
