# Supplementary material for: Green synthesis of binary FeOOH/Fe2O3 nanosized composite using Leucaena leucocephala seeds and their effect on mung bean under drought stress
Source: BMC Plant Biol. 2026 Mar 20;26:639. doi: 10.1186/s12870-026-08421-0 (PMC13064113; doi:10.1186/s12870-026-08421-0)
Supplement: Supplementary file 1 — Supplementary Material 1. [file 12870_2026_8421_MOESM1_ESM.pdf]

SCIENTIFIC SOLUTIONS

CERTIFICATE OF ENGLISH EDITING

This certificate confirms that the manuscript listed below was edited by an expert English editor with a PhD.

The following issues were corrected: grammar, spelling, punctuation, sentence structure, and phrasing.

Manuscript title

Green synthesis of binary FeOOH/Fe_2O_3 nanosized composite using Leucaena leucocephala seeds and their effect on mung bean under drought stress

Date issued

25 January 2026

Certificate Number

A07/2026

Cairo, Egypt

SCIENTIFIC SOLUTIONS

M. A. Ali
